# Genome-wide association and genotype by environment interactions for growth traits in U.S. Gelbvieh cattle

**DOI:** 10.1186/s12864-019-6231-y

**Published:** 2019-12-04

**Authors:** Johanna L. Smith, Miranda L. Wilson, Sara M. Nilson, Troy N. Rowan, David L. Oldeschulte, Robert D. Schnabel, Jared E. Decker, Christopher M. Seabury

**Affiliations:** 1 Department of Veterinary Pathobiology, Texas A&M University, College Station, 77843 USA; 2 Division of Animal Sciences, University of Missouri, Columbia, 65211 USA; 3 Genetics Area Program, University of Missouri, Columbia, 65211 USA; 4 Informatics Institute, University of Missouri, Columbia, 65211 USA

**Keywords:** GWAA, QTL, Genotype-by-environment interaction, Growth traits, Gelbvieh

## Abstract

**Background:**

Single nucleotide polymorphism (SNP) arrays have facilitated discovery of genetic markers associated with complex traits in domestic cattle; thereby enabling modern breeding and selection programs. Genome-wide association analyses (GWAA) for growth traits were conducted on 10,837 geographically diverse U.S. Gelbvieh cattle using a union set of 856,527 imputed SNPs. Birth weight (BW), weaning weight (WW), and yearling weight (YW) were analyzed using GEMMA and EMMAX (via imputed genotypes). Genotype-by-environment (GxE) interactions were also investigated.

**Results:**

GEMMA and EMMAX produced moderate marker-based heritability estimates that were similar for BW (0.36–0.37, SE = 0.02–0.06), WW (0.27–0.29, SE = 0.01), and YW (0.39–0.41, SE = 0.01–0.02). GWAA using 856K imputed SNPs (GEMMA; EMMAX) revealed common positional candidate genes underlying pleiotropic QTL for Gelbvieh growth traits on BTA6, BTA7, BTA14, and BTA20. The estimated proportion of phenotypic variance explained (PVE) by the lead SNP defining these QTL (EMMAX) was larger and most similar for BW and YW, and smaller for WW. Collectively, GWAAs (GEMMA; EMMAX) produced a highly concordant set of BW, WW, and YW QTL that met a nominal significance level (*P* ≤ 1e-05), with prioritization of common positional candidate genes; including genes previously associated with stature, feed efficiency, and growth traits (i.e., *PLAG1*, *NCAPG*, *LCORL*, *ARRDC3*, *STC2*). Genotype-by-environment QTL were not consistent among traits at the nominal significance threshold (*P* ≤ 1e-05); although some shared QTL were apparent at less stringent significance thresholds (i.e., *P* ≤ 2e-05).

**Conclusions:**

Pleiotropic QTL for growth traits were detected on BTA6, BTA7, BTA14, and BTA20 for U.S. Gelbvieh beef cattle. Seven QTL detected for Gelbvieh growth traits were also recently detected for feed efficiency and growth traits in U.S. Angus, SimAngus, and Hereford cattle. Marker-based heritability estimates and the detection of pleiotropic QTL segregating in multiple breeds support the implementation of multiple-breed genomic selection.

## Background

Growth traits are commonly recorded and used as selection criteria within modern beef cattle breeding programs and production systems; primarily because of their correlation with increased overall meat production and other economically important traits [1–4]. Some of the most commonly investigated growth traits include birth weight (BW), weaning weight (WW) and yearling weight (YW); with BW considered as both a production indicator, and a primary selection criterion for improving calving ease by reducing dystocia events [1, 2, 5–7]. Moreover, while previous studies have demonstrated that low estimated breeding values (EBVs) for BW are associated with reductions in both calf viability [6] and growth rates [5, 7], increased dystocia rates may also occur if sires with high EBVs for BW are used in conjunction with dams that possess small pelvic size. Therefore, modern beef breeding programs and production systems generally strive to increase calving ease, and maximize other growth-related traits such as WW and YW, particularly considering the known correlations between growth traits and other economically important carcass and reproductive traits [3, 5, 7].

Given the increasing economic importance of growth traits in beef cattle, a number of studies have sought to identify quantitative trait loci (QTL) influencing bovine body weight, growth, and aspects of stature, including both linkage studies and modern genome-wide association analyses [2, 8–13]. Several recent studies have also established moderate heritability estimates for bovine growth traits in U.S. beef cattle including BW, WW, and YW [14–17], with a number of relevant QTL and positional candidate genes identified to date, including orthologous genes that affect both human and bovine height [2, 18–22]. Notably, with the advent of the bovine genome assembly [23], the development of the Illumina Bovine SNP50 and 778K HD assays [23, 24], and more recently, the demonstrated ability to impute high density genotypes with high accuracy [25], an industry-supported research framework [26] has emerged that allows for very large-sample studies to be conducted without the costs associated with directly ascertaining high density genotypes (≥ 778K) for all study animals.

Herein, we used 10,837 geographically diverse U.S. Gelbvieh beef cattle and a union set of 856,527 (856K) imputed array variants to conduct GWAA with marker-based heritability estimates for BW, WW, and YW. Additionally, we used thirty-year climate data and K-means clustering to assign all Gelbvieh beef cattle to discrete U.S. climate zones for the purpose of estimating genotype-by-environment (GxE) interactions for BW, WW, and YW. This study represents the largest, high-density, single breed report to date with both standard GWAA and GxE GWAA for BW, WW, and YW. Additionally, we also evaluate the general concordance of GWAAs conducted using two popular methods (GEMMA; EMMAX) [27–29]. The results of this study are expected to positively augment current beef cattle breeding programs and production systems, particularly for U.S. Gelbvieh cattle, but also serve to highlight the increasing potential for eliciting economic impacts from industry-supported research frameworks that were developed for enhancing U.S. food security.

## Results and discussion

### Heritability estimates for BW, WW, and YW in U.S. Gelbvieh beef cattle

Herein, we used two approaches to generate marker-based heritability estimates for all investigated traits. Specifically, standardized relatedness matrices produced with GEMMA (*G*_*s*_) [27] and genomic relationship matrices (GRM) normalized via Gower’s centering approach and implemented in EMMAX [25, 28–30], were used to compare the chip or pseudo-heritability estimates for each investigated trait (Table 1). Notably, both approaches produced moderate heritability estimates with small standard errors for BW, WW, and YW; and heritability estimates for YW were highest among all investigated traits for U.S. Gelbvieh beef cattle. Moderate heritability estimates produced here using both approaches further support the expectation of positive economic gains resulting from the implementation of genomic selection [30].

**Table 1:** Variance component analysis with marker-based heritability estimates

Trait	GEMMA^a^ h^2^	GEMMA^a^ SE of h^2^	GEMMA^a^ V_g_	GEMMA^a^ V_e_	EMMAX^a^ h^2^	EMMAX^a^ SE of h^2^	EMMAX^a^ V_g_	EMMAX^a^ V_e_
BW	0.36	0.02	15.65	27.62	0.37	0.06	15.87	27.56
WW	0.27	0.01	712.07	1910.71	0.29	0.01	757.63	1881.68
YW	0.39	0.02	2751.21	4242.85	0.41	0.01	2902.56	4157.79

^**a**^ GEMMA chip heritability [27]; EMMAX pseudo-heritability [28, 29]

### GWAA for BW, WW, and YW in U.S. Gelbvieh beef cattle

**Fig. 1:**
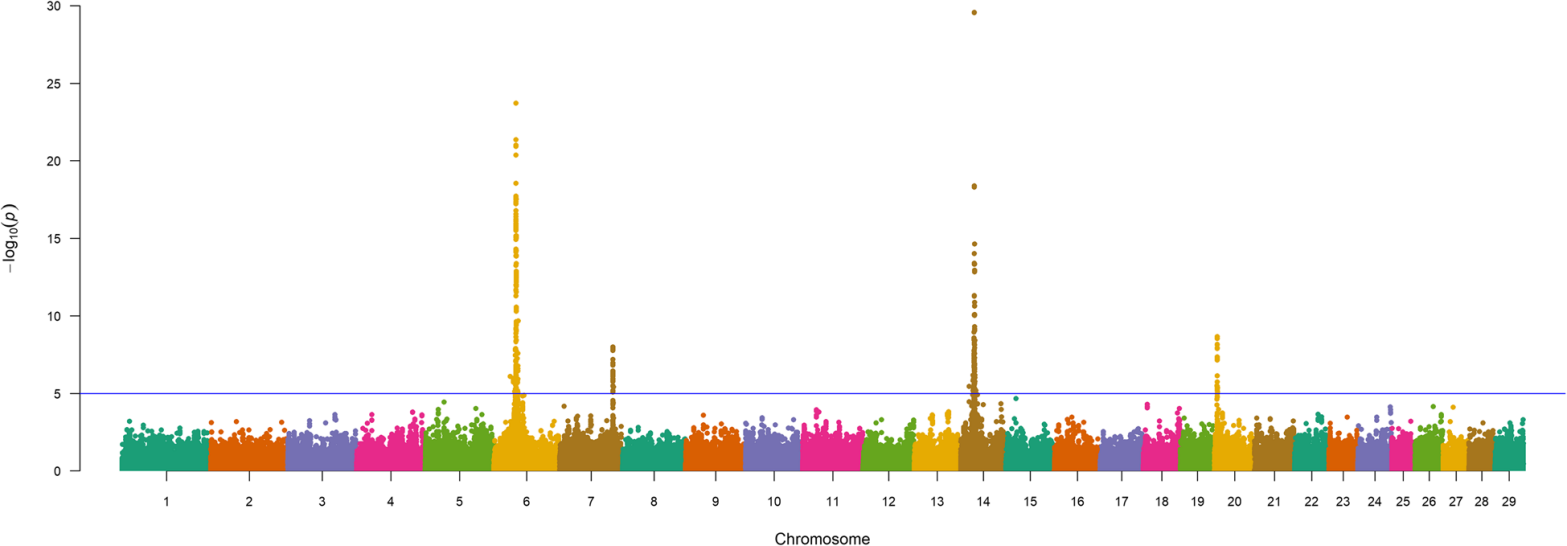
Birth weight (BW) QTL. Manhattan plot with GEMMA -log_10_ *P*-values. Lead and supporting SNPs for QTL represented at or above the blue line (*P* ≤ 1e-05; −log_10_ *P*-values ≥ 5.00) for n = 10,837 U.S. Gelbvieh beef cattle. A summary of all markers passing the nominal significance threshold [31] is presented in Table 2

The results of our 856K single-marker analyses for BW (GEMMA; EMMAX) [27–29] are shown in Fig. 1 and in Figure S1 (Additional File 1), with detailed summary data for QTL detected by GEMMA and EMMAX described in Table 2 and Table S1, respectively. A comparison of GEMMA and EMMAX results revealed a concordant set of QTL defined by lead SNPs (i.e., the most strongly associated SNP within a QTL region) which met a nominal significance threshold (*P* ≤ 1e-05) [31] (Table 2, Table S1, Additional File 1, Additional File 2). Specifically, QTL signals for BW were detected on BTA6, BTA7, BTA14, and BTA20 across both analyses (Table 2, Table S1, Additional File 1), and included an array of positional candidate genes generally involved in diverse aspects of mammalian growth and development (i.e., *CCSER1*, *ST18, RP1/XKR4, SLIT2, STC2, IBSP*) as well as bovine growth (i.e., *NCAPG*, *LCORL, KCNIP4, ARRDC3*), stature (i.e., *PLAG1*), and production traits (i.e., *IMPAD1*/*FAM110B, HERC6/PPM1K*) [2, 13, 14, 18, 21, 22, 30, 32–60]. Interestingly, the lead SNP defining the BW QTL detected on BTA14 (14_25 Mb) was located in *PLAG1*, thereby further supporting the involvement of this gene in various aspects of bovine growth and stature across breeds [2, 14, 18, 21, 30, 32–34]. Additionally, all but one (i.e., *NCAPG*, exon 9) of the lead SNPs for the detected Gelbvieh BW QTL (GEMMA, EMMAX) were noncoding variants (Table 2, Table S1, Additional File 1). Genomic inflation factors and correlation coefficients for *P*-values obtained from all BW analyses are shown in Tables S2-S3 (Additional File 1).

**Table 2:** Summary of QTL detected by GEMMA for BW in U.S. Gelbvieh beef cattle

Chr_Mb	MAF	-log_10_ *P*-value	Supporting SNPs	Positional Candidate Genes	Lead SNP Location	Scientific Precedence [reference]; organism; trait
*14_25*^*a*^	0.398	29.56	41	*PLAG1*	3’UTR	[2, 14, 18, 21, 30, 32–34]; Cattle; SimAngus mid-test metabolic weight association, carcass weight, stature, body weight and milk
*6_39*^*a*^	0.293	23.71	140	*NCAPG*	Exon^b^	[18, 21, 30, 35–39]; Cattle, chicken; stature, calving ease and growth traits association, SimAngus mid-test metabolic weight association, fetal growth, carcass trait association, average daily gain and daily feed intake, muscle mass
*14_26*^*a*^	0.396	14.63	33	*IMPAD1, FAM110B*	Intergenic	[30, 32, 34, 40]; Cattle; SimAngus mid-test metabolic weight association, carcass weight association, stature and body weight association, bone and cartilage system
*6_42*^*a*^	0.186	9.66	9	*KCNIP4*	Intron	[39, 41, 42]; Chicken, cattle, human; growth and muscle mass trait association, potassium channel activity
*14_24*^*a*^	0.244	8.93	35	*XKR4*	Intron	[2, 30, 43, 44]; Cattle; birth weight association, SimAngus mid-test metabolic weight association, growth trait association, feed intake and growth traits
*20_05*^*a*^	0.193	8.65	21	*LOC104975192, STC2*	Intergenic	[30, 45]; Cattle, mouse; mid-test metabolic weight in Hereford and SimAngus, developing and adult tissue maintenance, body size, related to post-natal growth
*7_93*^*a*^	0.283	8.00	30	*ARRDC3, LOC104972872*	Intergenic	[14, 22, 30, 46]; Cattle; body and carcass weight association, calving ease, average daily gain in Hereford, growth and muscularity, birth weight, weaning weight, yearling weight, and ribeye area in Angus
*6_38*^*a*^	0.053	7.90	23	*IBSP, LOC104972726*	Intergenic	[13, 47–49]; Cattle, mouse, human; yearling weight association, bone formation and remodeling, cellular proliferation, milk fat and protein association
*6_41*^*a*^	0.407	7.25	5	*LOC782905, SLIT2*	Intergenic	[39, 49–53]; Cattle, chicken, human; milk fat and protein association, organ and muscle weight, development of central nervous system, tumor suppressor activity
*14_23*^*a*^	0.467	6.19	3	*ST18*	Intron	[54]; Human; regulation of apoptosis and inflammatory response
*6_34*^*a*^	0.039	5.98	8	*LOC104972717, LOC526089*	Intergenic	NA
*6_40*^*a*^	0.304	5.25	2	*LCORL, LOC782905*	Intergenic	[18, 21, 37–39, 50, 55, 56]; Cattle, sheep; stature, muscle and organ growth, feed intake and gain association, growth and carcass traits, skeletal growth and muscle mass

^a^ Indicates QTL was detected in EMMAX analysis

^b^ Indicates a predicted nonsynonymous mutation Ile➔Met, exon 9

**Fig. 2:**
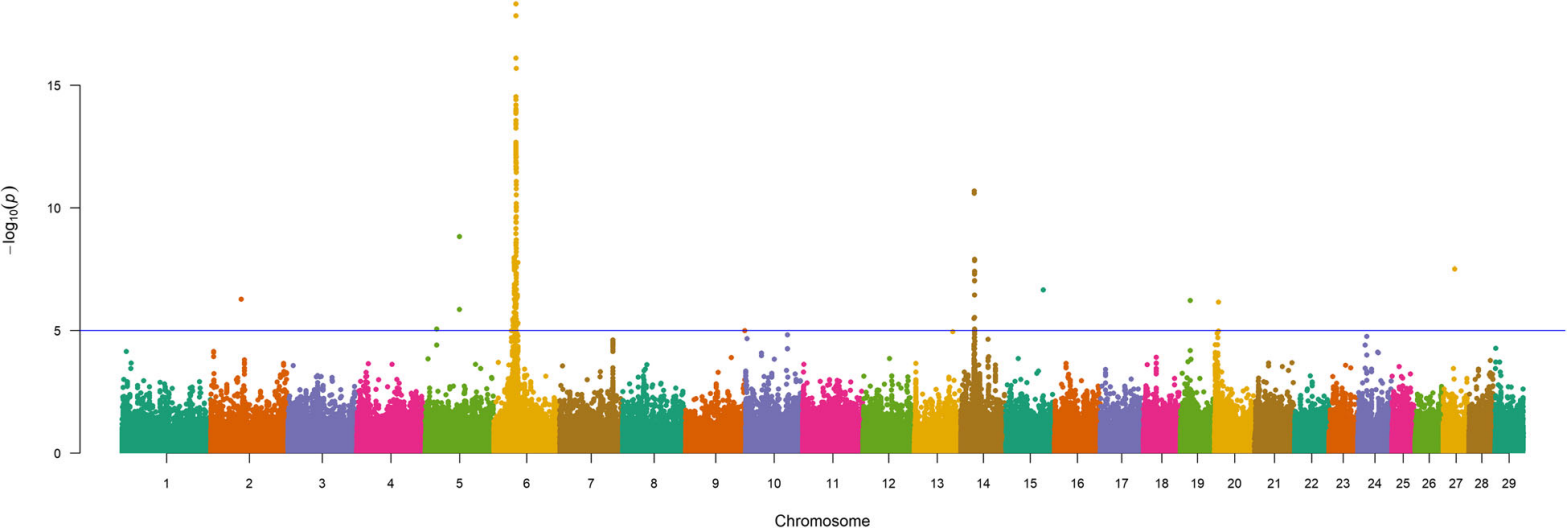
Weaning weight (WW) QTL. Manhattan plot with GEMMA -log_10_ *P*-values. Lead and supporting SNPs for QTL represented at or above the blue line (*P* ≤ 1e-05; −log_10_ *P*-values ≥ 5.00) for n = 10,837 U.S. Gelbvieh beef cattle. A summary of all markers passing the nominal significance threshold [31] is presented in Table 3

Single-marker analyses (856K) for WW in U.S. Gelbvieh beef cattle (GEMMA; EMMAX) revealed several of the same QTL detected for BW (Table 3, Fig. 2, Table S4, Figure S2, Additional File 1), thus providing statistical support for pleiotropic QTL located on BTA6 (i.e., *NCAPG*, *CCSER1*, *KCNIP4*, *HERC6/PPM1K*, *LOC782905/SLIT2*, *LOC100336621/LOC104972717*) as well as BTA14 (i.e., *PLAG1*, *XKR4*, *IMPAD1/FAM110B*). The lead SNPs for Gelbvieh BW and WW QTL detected on BTA20 (20_05 Mb) suggested proximal but independent causal mutations, thus implicating the potential involvement of at least three positional candidate genes (*LOC104975192*/*STC2*, *ERGIC1*). A detailed summary of lead and supporting SNPs for pleiotropic QTL is provided in Additional File 2. Beyond evidence for pleiotropy, four additional Gelbvieh WW QTL were also detected on BTA5 (5_60 Mb), BTA6 (6_31 Mb, 6_37 Mb) and BTA28 (28_37 Mb; Table 3, Fig. 2, Table S4, Figure S2, Additional File 1). Among the additional QTL detected, several positional candidate genes have been implicated in aspects of development (*UNC5C*, *SNCA*/*GPRIN3*) and immune function (*SH2D4B*) [61–67]. An investigation of all lead SNPs for the detected Gelbvieh WW QTL revealed 13 noncoding variants and one nonsynonymous variant (Table 3, Table S4, Additional File 1). Genomic inflation factors and correlation coefficients for *P*-values obtained from all WW analyses are presented in Tables S2 and S3 (Additional File 1).

**Table 3:** Summary of QTL detected by GEMMA for WW in U.S. Gelbvieh beef cattle

Chr_Mb	MAF	-log_10_ *P*-value	Supporting SNPs	Positional Candidate Genes	Lead SNP Location	Scientific Precedence [reference]; organism; trait
*6_39*^*a*^	0.289	18.32	107	*NCAPG*	Exon^b^	[18, 21, 30, 35–39]; Cattle, chicken; stature, calving ease and growth traits association, SimAngus mid-test metabolic weight association, fetal growth, carcass trait association, average daily gain and daily feed intake, muscle mass
*14_25*^*a*^	0.398	10.69	2	*PLAG1*	3’UTR	[2, 14, 18, 21, 30, 32–34]; Cattle; SimAngus mid-test metabolic weight association, carcass weight, stature, body weight and milk
*5_60*^*a*^	0.046	8.83	2	*LOC527216, LOC788998*	Intergenic	NA
*6_36*^*a*^	0.214	7.95	29	*CCSER1*	Intron	[14, 60]; Cattle, human; body and carcass weight association, regulator of mitosis
*14_26*^*a*^	0.415	7.90	11	*IMPAD1, FAM110B*	Intergenic	[30, 32, 34, 40]; Cattle; SimAngus mid-test metabolic weight association, carcass weight association, stature and body weight association, bone and cartilage system
*6_42*^*a*^	0.340	7.77	3	*KCNIP4*	Intron	[39, 41, 42]; Chicken, cattle, human; growth and muscle mass trait association, potassium channel activity
*6_38*^*a*^	0.220	7.70	9	*HERC6, PPM1K*	Intergenic	[49, 58, 59]; Cattle; milk, fat, and protein yield, metabolic processes, feed efficiency association
*6_41*^*a*^	0.238	6.46	4	*LOC782905, SLIT2*	Intergenic	[39, 49–53]; Cattle, chicken, human; milk fat and protein association, organ and muscle weight, development of central nervous system, tumor suppressor activity
*6_37*^*a*^	0.325	5.97	5	*SNCA, GPRIN3*	Intergenic	[61–64]; Human, goat, equine; neurological regulation, milk and meat associations, tendon tissue association
*6_34*^*a*^	0.295	5.36	4	*LOC100336621, LOC104972717*	Intergenic	NA

^a^ Indicates QTL was detected in EMMAX analysis

^b^ Indicates a predicted nonsynonymous mutation Ile➔Met, exon 9

**Fig. 3:**
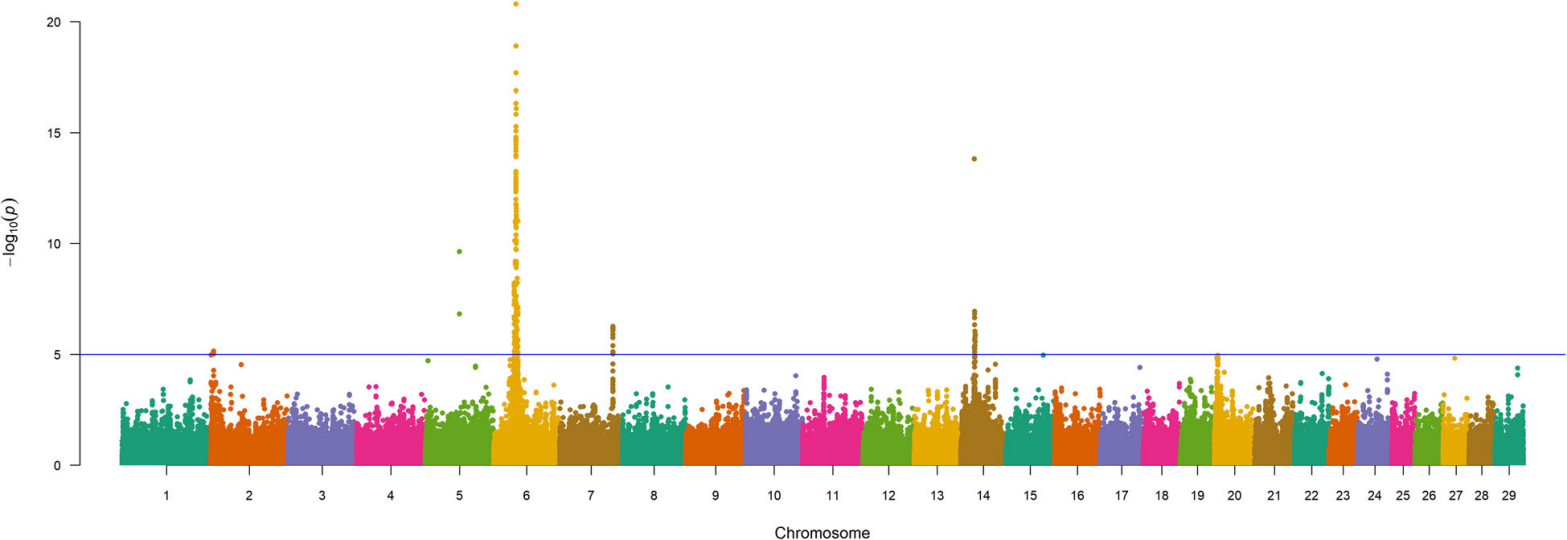
Yearling weight (YW) QTL. Manhattan plot with GEMMA -log_10_ *P*-values. Lead and supporting SNPs for QTL represented at or above the blue line (*P* ≤ 1e-05; −log_10_ *P*-values ≥ 5.00) for n = 10,837 U.S. Gelbvieh beef cattle. A summary of all markers passing the nominal significance threshold [31] is presented in Table 4

Consistent with our analyses of BW and WW, our single-marker analyses (856K) for YW in U.S. Gelbvieh beef cattle again revealed evidence for pleiotropic QTL located on BTA6 and BTA14 (Table 4, Fig. 3, Table S5, Figure S3, Additional File 1). Specifically, the results obtained from our analyses of BW, WW, and YW revealed some common QTL signals for all investigated traits on BTA6 (6_36 Mb, 6_38 Mb, 6_39 Mb, 6_41 Mb, 6_42 Mb) and BTA14 (14_24 Mb, 14_25 Mb, 14_26 Mb). Likewise, the lead SNPs defining these QTL also resulted in the prioritization of the same positional candidate genes on BTA6 (i.e., *LCORL*, *KCNIP4*, *HERC6*/*PPM1K*, *SLIT2*, *CCSER1*) and BTA14 (i.e., *PLAG1*, *IMPAD1*/*FAM110B, RP1/XKR4*). Together with pleiotropic signals on BTA6 and BTA14, eight additional YW QTL were also detected; including one QTL (7_93 Mb) that was also found to influence Gelbvieh BW (Table 4, Table S5, Additional File 1). Positional candidate genes for these QTL have been implicated in diverse aspects of growth and development as well as bovine production traits (i.e., *SNCA*/*GPRIN3*, *SLIT2*, *NSMAF*, *LOC101905238*/*ARRDC3*), bovine milk traits (i.e., *PPARGC1A*), and chromatin modification (i.e., *IWS1*) [68–71]. Relevant to YW, it should also be noted that several of the pleiotropic QTL detected for U.S. Gelbvieh in this study have also been detected for mid-test metabolic weight in U.S. SimAngus beef cattle (6_39 Mb, 14_24 Mb, 14_25 Mb, 14_26 Mb) [30]. Moreover, Gelbvieh QTL (BW, YW) detected on BTA14 and BTA7 have also been detected for Angus residual feed intake (14_27 Mb), and Hereford average daily gain (7_93 Mb) [30]. An investigation of all lead SNPs for the detected Gelbvieh YW QTL revealed 16 noncoding variants (Table 4, Table S5, Additional File 1). Genomic inflation factors and correlation coefficients for *P*-values obtained from all YW analyses are shown in Tables S2-S3 (Additional File 1).

**Table 4:** Summary of QTL detected by GEMMA for YW in U.S. Gelbvieh beef cattle

Chr_Mb	MAF	-log_10_ *P*-value	Supporting SNPs	Positional Candidate Genes	Lead SNP Location	Scientific Precedence [reference]; organism; trait
*6_39*^*a*^	0.305	20.81	103	*LCORL*	Intron	[18, 21, 30, 37–39, 55, 56]; Cattle, sheep; stature, SimAngus mid-test metabolic weight association, muscle and organ growth, feed intake and gain association, growth and carcass traits, skeletal growth and muscle mass
*14_25*^*a*^	0.399	13.82	3	*PLAG1*	3’UTR	[2, 14, 18, 21, 30, 32–34]; Cattle; SimAngus mid-test metabolic weight association, carcass weight, stature, body weight and milk
*6_38*^*a*^	0.222	11.00	20	*HERC6, PPM1K*	Intergenic	[49, 58, 59]; Cattle; milk, fat, and protein yield, metabolic processes, feed efficiency association
*6_42*^*a*^	0.344	11.00	11	*KCNIP4*	Intron	[39, 41, 42]; Chicken, cattle, human; growth and muscle mass trait association, potassium channel activity
*6_37*^*a*^	0.330	10.12	8	*SNCA, GPRIN3*	Intergenic	[61–64]; Human, goat, equine; neurological regulation, milk and meat associations, tendon tissue association
*5_60*^*a*^	0.042	9.62	2	*LOC527216, LOC788998*	Intergenic	NA
*6_41*^*a*^	0.247	8.44	6	*SLIT2*	Intron	[39, 49–53]; Cattle, chicken, human; milk fat and protein association, organ and muscle weight, development of central nervous system, tumor suppressor activity
*6_36*^*a*^	0.227	8.23	20	*CCSER1*	Intron	[14, 60]; Cattle, human; body and carcass weight association, regulator of mitosis
*14_26*^*a*^	0.357	6.94	12	*IMPAD1, FAM110B*	Intergenic	[30, 32, 34, 40]; Cattle; SimAngus mid-test metabolic weight association, carcass weight association, stature and body weight association, bone and cartilage system
*7_93*^*a*^	0.286	6.23	14	*LOC101905238,* *ARRDC3*	Intergenic	[14, 22, 30, 46]; Cattle; body and carcass weight association, calving ease, average daily gain in Hereford, growth and muscularity, birth weight, weaning weight, yearling weight, and ribeye area in Angus
*6_40*^*a*^	0.109	6.21	11	*LOC782905, SLIT2*	Intergenic	[39, 49–53]; Cattle, chicken, human; milk fat and protein association, organ and muscle weight, development of central nervous system, tumor suppressor activity
*14_27*^*a*^	0.348	6.04	6	*NSMAF*	Intron	[30, 68]; Cattle, human; Angus residual feed intake association, immune system response
*2_05*	0.497	5.15	3	*IWS1*	Intron	[69]; Human; chromatin modification, histone chaperone, maintenance of virus latency

^a^ Indicates QTL was detected in EMMAX analysis

### GxE GWAA for BW, WW, and YW in U.S. Gelbvieh beef cattle

**Fig. 4:**
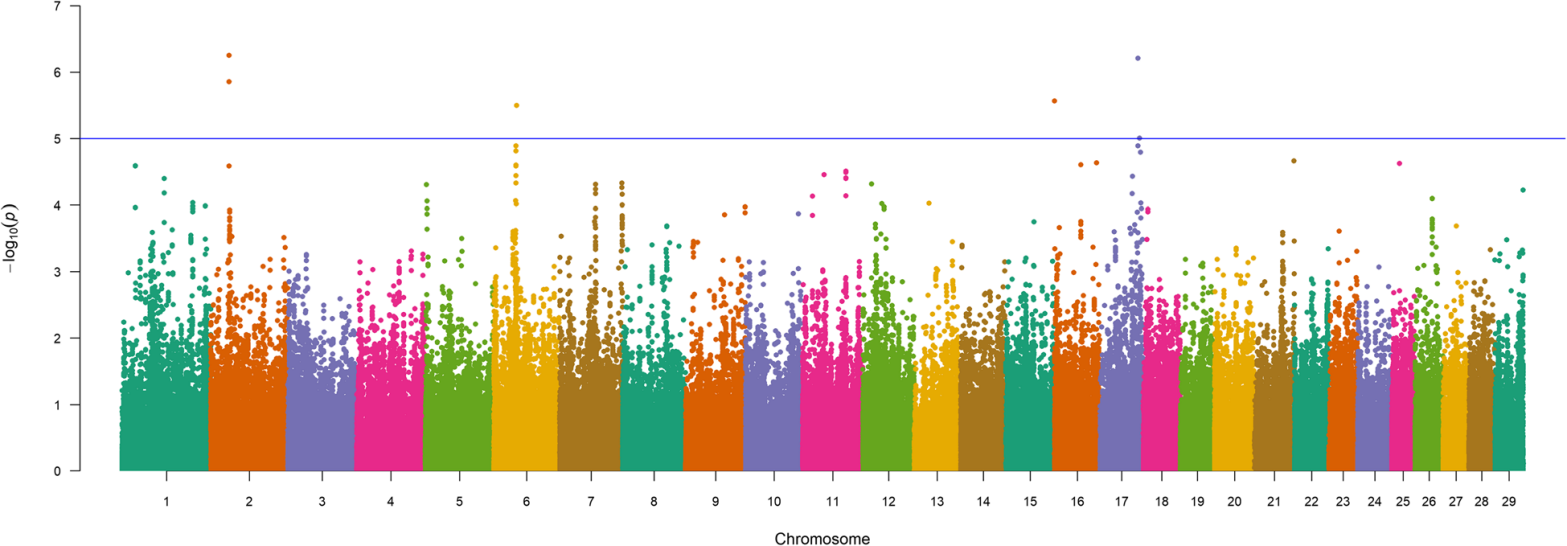
Birth weight genotype-by-environment (BW GxE) QTL. Manhattan plot with GEMMA -log_10_ *P*-values. Lead and supporting SNPs for QTL represented at or above the blue line (*P* ≤ 1e-05; −log_10_ *P*-values ≥ 5.00) for n = 10,837 U.S. Gelbvieh beef cattle. A summary of all markers passing the nominal significance threshold [31] is presented in Table 5

To investigate the potential for significant GxE interactions in relation to BW, WW, and YW in U.S. Gelbvieh beef cattle, we conducted six additional single-marker (856K) analyses using both GEMMA and EMMAX [27–29]. For all analyses, we included a variable for Gelbvieh geographic zone, which was generated via K-means clustering using thirty-year U.S. climate data, and treated as an interaction term (See Methods). Notably, a BW GxE QTL detected on BTA2 (2_32 Mb; lead SNP is intergenic) revealed multiple biologically relevant positional candidate genes, including *GRB14*, which has been shown to regulate insulin in mice [72], and *FIGN*, which has been associated with plasma folate levels in humans (Fig. 4, Table 5, Additional File 2) [73]. Importantly, maternal folate levels have been shown to influence human birthweight [74], and a role for insulin regulation in bovine feed efficiency and growth traits has also been described [30]. Beyond BTA2, BW GxE QTL were also detected on BTA17 (17_66 Mb) and BTA13 (13_67 Mb). Positional candidate genes for these QTL have been implicated in the removal of uracil residues from DNA and apoptosis (*UNG*) as well as human obesity (*CTNNBL1*) (Fig. 4, Table 5, Figure S4, Table S6, Additional File 1) [75, 76]. Examination of the lead SNPs for all GxE QTL detected for Gelbvieh BW (Table 5, Table S6, Additional File 1, Additional File 2) revealed three noncoding variants, which is suggestive of quantitative (i.e., regulatory) effects. Genomic inflation factors and correlation coefficients for *P*-values obtained from all GxE BW analyses are shown in Tables S2-S3 (Additional File 1).

**Table 5:** Summary of GxE QTL detected by GEMMA for BW in U.S. Gelbvieh beef cattle

Chr_Mb	MAF	-log_10_ *P*-value	Supporting SNPs	Positional Candidate Genes	Lead SNP Position	Scientific Precedence [reference]; organism; trait
*2_32*	0.105	6.25	2	*GRB14, FIGN*	Intergenic	[72–74]; Mouse, human; insulin receptor related to growth and metabolism, folic acid association with impact on BW
*17_66*	0.026	6.21	2	*UNG*	Intron	[75]; Human; DNA maintenance

**Fig. 5:**
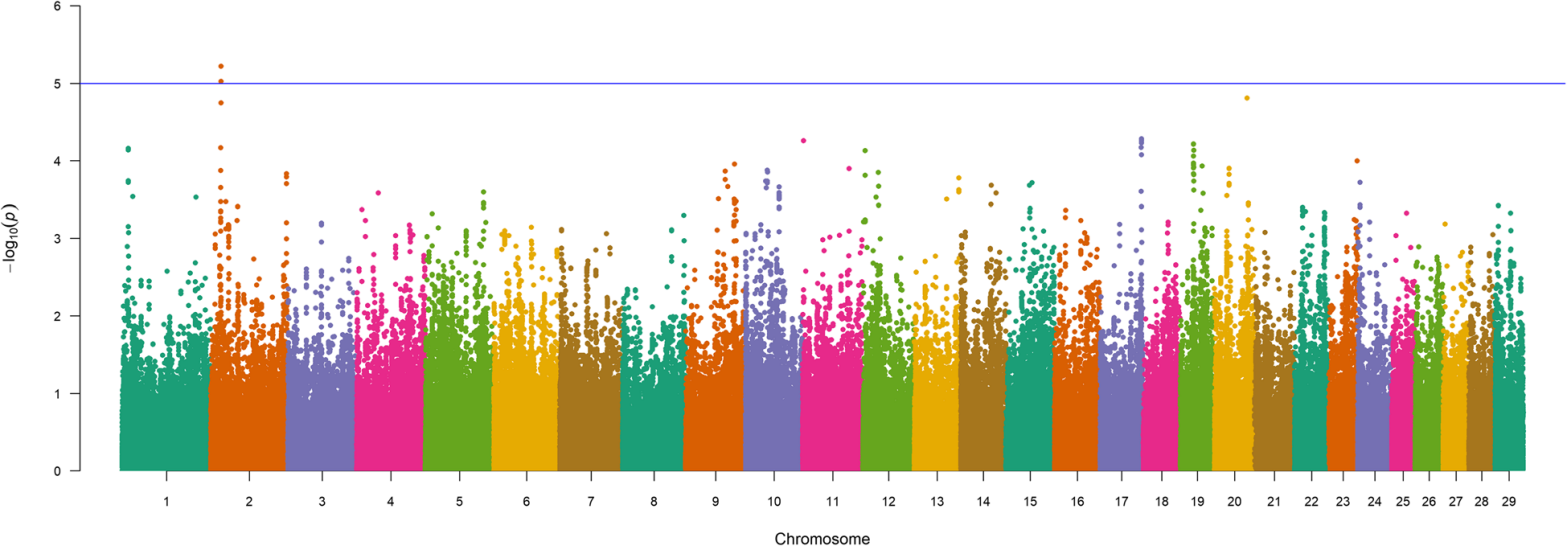
Weaning weight genotype-by-environment (WW GxE) QTL. Manhattan plot with GEMMA -log_10_ *P*-values. Lead and supporting SNPs for QTL represented at or above the blue line (*P* ≤ 1e-05; −log_10_ *P*-values ≥ 5.00) for n = 10,837 U.S. Gelbvieh beef cattle. A summary of all markers passing the nominal significance threshold [31] is presented in Table 6

Our analyses (GEMMA, EMMAX) to evaluate the potential for significant GxE interactions with respect to WW in U.S. Gelbvieh beef cattle produced evidence for one GxE QTL on BTA2 (2_18 Mb) which was only detected by GEMMA, and included relatively few supporting SNPs (*P* ≤ 1e-05, Table 6; Fig. 5, Figure S5, Additional File 1). The lead SNP defining this QTL was located in exon 304 of *TTN*, and encoded a nonsynonymous variant (Table 6, Fig. 5, Additional File 2). Interestingly, *TTN* is known to function as a myofilament system for skeletal and cardiac muscle, with mouse M-line deficient knockouts resulting in sarcomere disassembly as well as muscle atrophy and death [77–79].

**Table 6:** Summary of GxE QTL detected by GEMMA for WW in U.S. Gelbvieh beef cattle

Chr_Mb	MAF	-log_10_ P-value	Supporting SNPs	Positional Candidate Genes	Lead SNP Location	Scientific Precedence [reference]; organism; trait
*2_18*	0.012	5.22	2	*TTN*	Exon^a^	[77–79]; Rabbit, rat, human; aids in myofibrillar assembly, positioning of myosin filaments in muscle, coordinates multiple signaling pathways for gene activation, protein folding, quality control and degradation, heart disease relation

^a^ Indicates a predicted nonsynonymous mutation Arg➔Gln, exon 304

**Fig. 6:**
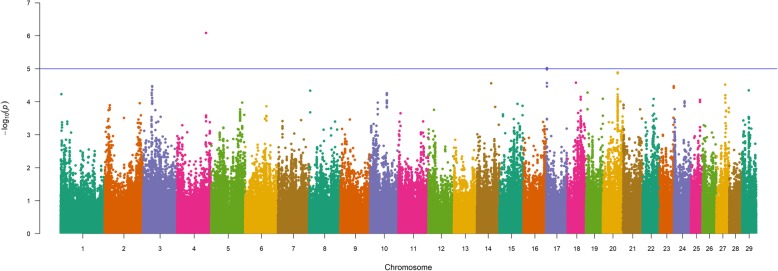
Yearling weight genotype-by-environment (YW GxE) QTL. Manhattan plot with GEMMA -log_10_ *P*-values. Lead and supporting SNPs for QTL represented at or above the blue line (*P* ≤ 1e-05; −log_10_ *P*-values ≥ 5.00) for n = 10,837 U.S. Gelbvieh beef cattle. A summary of all markers passing the nominal significance threshold [31] is presented in Table 7

Analyses (GEMMA; EMMAX) to evaluate the potential for significant GxE interactions with respect to YW in U.S. Gelbvieh beef cattle revealed two GxE QTL with three positional candidate genes (*LRAT*/*LOC101904475*/*FGG*) on BTA17 (17_03 Mb), and one positional candidate gene on BTA5 (*PHF21B* at 116 Mb; *P* ≤ 1e-05, Table 7, Fig. 6, Table S7, Figure S6, Additional File 1, Additional File 2). The signal on BTA17 (i.e., GEMMA lead SNP in Intron 4 of *LOC101904475* and supporting SNPs) was replicated by EMMAX (Figure S6, Additional File 1); but at a less stringent significance threshold (i.e. *P* < 6e-04). Notably, while the function of *LOC101904475* remains unclear, *LRAT* is known to catalyze esterification of retinol (i.e., from Vitamin A) [80], and Vitamin A has been shown to promote growth in beef cattle as well as humans [81–83]. However, *FGG* is also an intriguing candidate, as fibrinogen has been shown to constrict blood vessels [84]. This vasoconstriction may alter the ability to cope with heat stress, but in the context of cattle production, the relationship between vasoconstriction and fescue toxicosis is perhaps more noteworthy. Fescue toxicosis is the result of ergot alkaloids produced by the endophytic fungus in fescue forage [85], especially the Kentucky 31 variety. One of the major symptoms of fescue toxicosis is vasoconstriction, thus variation in *FGG* expression levels may potentially alter cattle’s innate degree of vasoconstriction; perhaps further complicating both fescue toxicosis and heat stress. The other interesting positional candidate gene on BTA5 (*PHF21B*) is known to be involved in the modulation of stress responses, and the regulation of cellular division [86, 87].

**Table 7:** Summary of GxE QTL detected by GEMMA for YW in U.S. Gelbvieh beef cattle

Chr_Mb	MAF	-log_10_ *P*-value	Supporting SNPs	Positional Candidate Genes	Lead SNP Location	Scientific Precedence [reference]; organism; trait
*17_03*	0.328	5.02	2	*LRAT*, *LOC101904475*, *FGG*	Intron	[80–85]; Mouse, cattle, human, rat; retinal development, muscular growth and fiber composition, vitamin A regulation, vascular constriction

## Conclusions

Herein, we present evidence for pleiotropic QTL influencing BW, WW, and YW in U.S. Gelbvieh beef cattle, and further confirm the involvement of *PLAG1* in various aspects of bovine growth and stature across breeds [2, 14, 18, 21, 30, 32–34]. Additionally, we also present compelling evidence for QTL segregating in multiple breeds; with at least seven U.S. Gelbvieh growth QTL that were also detected for feed efficiency and growth traits in U.S. Angus, SimAngus, and Hereford beef cattle [30]. Despite the involvement of major genes such as *NCAPG*, *PLAG1* and *LCORL*, more of the phenotypic variance in Gelbvieh BW, WW, and YW was explained by many other genome-wide loci (See Additional File 1, Additional File 2). Moreover, we demonstrate that most of the Gelbvieh QTL are detectable by two different large-sample analyses (GEMMA; EMMAX). However, some discordant QTL detected by the GxE GWAAs can also be attributed to differences in the model specifications for these analyses, as implemented by GEMMA and EMMAX (See Methods). While relatively few GxE QTL were detected, the identified GxE QTL harbor physiologically meaningful positional candidates. Moreover, the results of this study demonstrate that imputation to a union set of high-density SNPs (i.e., 856K) for use in large-sample analyses can be expected to facilitate future discoveries at a fraction of the cost associated with direct genotyping, which also underscores the present impact of genomic tools and resources developed by the domestic cattle research community.

## Methods

Cattle phenotypes were received from the American Gelbvieh Association (pre-adjusted for age of animal [i.e. 205-day weight for WW] and age of dam as per breed association practice), and corresponding genotypes were transferred from their service provider Neogen GeneSeek. For GWAA analyses, the phenotypes were pre-adjusted for sex and contemporary group consisting of 5-digit breeder zip-code, birth year, and birth season (Spring, Summer, Fall, and Winter) using the mixed.solve() function from the rrBLUP package v4.4 [88] in R v3.3.3 [89].

To group individuals into discrete climate zones, K-means clustering was performed on three continuous climate variables. Thirty-year normal values for temperature, precipitation, and elevation were drawn from the PRISM climate dataset [90]. Each one km square of the continental United States was assigned to one of nine climate zones using K-means clustering implemented in the RStoolbox R package [91, 92]. The optimal number of zones was identified using the pamk function from the R package fpc [93]. Individuals were assigned to zones based on the zip code of their breeder as recorded in the American Gelbvieh Association herdbook.

Quality control was performed on genotypes for 13,166 Gelbvieh individuals using PLINK 1.9 [94]. Individuals with call rates < 0.90 were removed on an assay-by-assay basis (For assay information see Additional File 3). Variants with call rates < 0.90 or Hardy-Weinberg Equilibrium (HWE) *P*-values <1e-20 were also removed. For this analysis, only autosomal chromosomes were analyzed. After filtering, genotypes for the 12,422 individuals that remained were merged using PLINK and then phased using EagleV2.4 [95]. Genotypes inferred by Eagle were removed with bcftools [96]. Imputation was performed with IMPUTE2 [97] using the “merge_ref_panels” flag. This allowed the phased haplotypes for 315 individuals genotyped on the Illumina HD (Illumina, San Diego, CA) and 559 individuals genotyped on the GGP-F250 (GeneSeek, Lincoln, NE) to be recursively imputed and treated as reference haplotypes. These reference haplotypes were used to impute the remaining 11,598 low-density genotypes from various assays (Additional File 3) to the shared number of markers between the two high-density research chips. The resulting dataset consisted of 12,422 individuals with 856,527 markers each (UMD3.1). To account for uncertainty in imputation, IMPUTE2 reports dosage genotypes. Hard-called genotypes were inferred from dosages using PLINK. When making hard-calls, PLINK treats genotypes with uncertainty > 0.1 as missing. This resulted in a hard-called dataset of 856,527 variants, which includes genotypes set as missing. Prior to the execution of all GWAAs (GEMMA; EMMAX), we filtered the Gelbvieh samples and all SNP loci as follows: Gelbvieh sample call rate filtering (< 90% call rate excluded); thereafter SNP filtering by call rate (> 15% missing excluded), MAF (< 0.01 excluded), polymorphism (monomorphic SNPs excluded), and HWE (excludes SNPs with HWE *P* < 1e-50), which resulted in 618,735 SNPs. Additionally, prior to all GWAAs (GEMMA; EMMAX) hard-called genotypes were numerically recoded as 0, 1, or 2, based on the incidence of the minor allele. Missing hard-called genotypes (i.e., that met our filtering criteria) were modeled as the SNP’s average value (0, 1, or 2) across all samples.

Using the numerically recoded hard-called genotypes and the adjusted Gelbvieh phenotypes, we employed GEMMA to conduct univariate linear mixed model GWAAs where the general mixed model can be specified as *y* = *Wα* + *xβ* + *u* + *ϵ*; where *y* represents a *n*-vector of quantitative traits for *n*-individuals, *W* is an *n* x *c* matrix of specified covariates (fixed effects) including a column of 1s, *α* is a *c*-vector of the corresponding coefficients including the intercept, *x* represents an *n*-vector of SNP genotypes, *β* represents the effect size of the SNP, *u* is an *n*-vector of random effects, and *ϵ* represents an *n*-vector of errors [27]. Moreover, it should also be noted that *u* ∼ *MVN*_*n*_(0, *λτ*^−1^Κ) and *ϵ* ∼ *MVN*_*n*_(0, *λτ*^−1^Ι_*n*_), where *MVN* denotes multivariate normal distribution, *λτ*^−1^ is the variance of the residual errors, *λ* is the ratio between the two variance components, Κ is a known *n* x *n* relatedness matrix, and Ι_*n*_ represents an *n* x *n* identity matrix [27]. Using this general approach, GEMMA evaluated the alternative hypothesis for each SNP (*H*_1_ : *β* ≠ 0) as compared to the null (*H*_0_ : *β* = 0) by performing a likelihood ratio test with maximum likelihood estimates (−lmm 2) as follows:

${D}_{lrt}=2\mathit{\log}\frac{l_1\left(\hat{\lambda}1\right)}{l_0\left(\hat{\lambda}0\right)}$, with *l*_1_ and *l*_0_ being the likelihood functions for the null and alternative models, respectively, where $\hat{\lambda}$_0_ and $\hat{\lambda}$_1_ represent the maximum likelihood estimates for the null and the alternative models, respectively, and where *P*-values come from a ${\mathcal{X}}^2$ distribution, as previously described [27]. Herein, the only fixed-effect covariate specified for all GWAAs was an environmental variable (geographic zone for each individual). For all GxE GWAAs (−gxe command), the environmental variable (geographic zone for each individual) was treated as an interaction term, where the resulting *P*-values represent the significance of the genotype x environment interaction. Specifically, for the GxE GWAAs in GEMMA, the model is specified as *y* = *Wα* + *x*_*snp*_*β*_*snp*_ + *x*_*env*_*β*_*env*_ + *x*_*snp*_ × *x*_*env*_*β*_*snp* × *env*_ + *u* + *ϵ*; where *y* represents a *n*-vector of quantitative traits for *n*-individuals, *W* is an *n* x *c* matrix of specified covariates (fixed effects) including a column of 1s, *α* is a *c*-vector of the corresponding coefficients including the intercept, *x*_*snp*_ represents an *n*-vector of SNP genotypes, *β*_*snp*_ represents the effect size of the SNP, *x*_*env*_ represents an *n*-vector of environmental covariates, *β*_*env*_ represents the fixed effect of the environment, *β*_*snp* × *env*_ is the interaction between SNP genotype and environment, *u* is an *n*-vector of random effects, and *ϵ* represents an *n*-vector of errors. GEMMA evaluated the alternative hypothesis for each interaction (*H*_1_ : *β*_*snp* × *env*_ ≠ 0) as compared to the null (*H*_0_ : *β*_*snp* × *env*_ = 0). Marker-based relatedness matrices (*G*_*s*_) relating instances of the random effect specified to each of the growth phenotypes among all genotyped cattle were used to estimate the proportion of variance explained (PVE) by the hard-called genotypes in GEMMA, which is also commonly referred to as the “chip heritability” [27, 98]. For all investigated traits, single-marker *P*-values obtained from GEMMA (−lmm 2, −gxe) were used to generate Manhattan plots in R (manhattan command) and QTL were defined by ≥ 2 SNP loci with MAF ≥ 0.01 (i.e., a lead SNP plus at least one additional supporting SNP within 1 Mb) which also met a nominal significance threshold (*P* ≤ 1e-05) [30, 31].

Using hard-called genotypes and the adjusted Gelbvieh phenotypes, we performed a second set of GWAAs using a mixed linear model with variance component estimates, as implemented by EMMAX [28–30, 99–101]. Briefly, the general mixed model used in this approach can be specified as: *y* = *Xβ* + *Zu* + *ϵ*, where *y* represents a *n* × 1 vector of phenotypes, *X* is a *n* × *q* matrix of fixed effects, *β* is a *q* × 1 vector representing the coefficients of fixed effects, and *Z* is a *n* × *t* matrix relating the random effect to the phenotypes of interest [30, 99–101]. Herein, we must assume that $Var(u)={\sigma}_g^2K$ and $Var\left(\epsilon \right)={\sigma}_e^2I$, such that $Var(y)={\sigma}_g^2 ZK{Z}^{\prime }+{\sigma}_e^2I$, however, in this study *Z* represents the identity matrix *I*, and *K* represents a kinship matrix of all Gelbvieh samples with hard-called genotypes. Moreover, to solve the mixed model equations using a generalized least squares approach, we must estimate the variance components (${\sigma}_g^2$ and ${\sigma}_e^2$) as previously described [28–30, 99, 100]. For this study, we estimated the variance components using the REML-based EMMA approach [29], with stratification accounted for and controlled using the genomic relationship matrix [25, 30], as computed from the Gelbvieh hard-called genotypes. Moreover, the only fixed-effect covariate specified for all GWAAs was an environmental variable (geographic zone for each individual). For all EMMAX GxE GWAAs utilizing hard-called genotypes, we used an implementation of EMMAX [29, 102] where interaction-term covariates may be specified; with the environmental variable (geographic zone for each individual) specified as the interaction term. The basis of this approach is rooted in full versus reduced model regression [99], where interaction-term covariates are included in the model as follows: each specified interaction-term covariate serves as one reduced-model covariate; each specified interaction-term covariate is also multiplied, element by element, with each SNP predictor (i.e., *SNP* × *geographic zone*) to create an interaction term to be included in the full model. Specifically, given *n* measurements of a Gelbvieh growth phenotype that is influenced by *m* fixed effects and *n* instances of one random effect, with one or more GxE effects (*e*) whereby the interaction is potentially with one predictor variable, we model this using a full and a reduced model. The full model can be specified as *y* = *X*_*c*_*β*_*kc*_ + *X*_*i*_*β*_*ki*_ + *X*_*k*_*β*_*kp*_ + *X*_*ip*_*β*_*ip*_ + *u*_*full*_ + *ϵ*_*full*_, and the reduced model as *y* = *X*_*c*_*β*_*krc*_ + *X*_*i*_*β*_*kri*_ + *X*_*k*_*β*_*rkp*_ + *u*_*reduced*_ + *ϵ*_*reduced*_, where *y* is an *n*-vector of observed phenotypes, *X*_*c*_ is an *n* × *m* matrix of *m* fixed-effect covariates, *X*_*i*_ is an *n* × *e* matrix of *e* fixed terms being tested for GxE interactions, *X*_*k*_ is an *n*-vector containing the covariate or predictor variable that may be interacting, and *X*_*ip*_ is an *n* × *e* matrix containing the *e* interaction terms created by multiplying the columns of *X*_*i*_ element-by-element with *X*_*k*_. All of the *β* terms correspond to the *X* terms as written above, and to the full or the reduced model, as specified, with *u* and *ϵ* representing the random effect and error terms, respectively. Like the EMMAX method without interactions [28, 29], we approximate this by finding the variance components once, using the parts of the above equations that are independent of *X*_*k*_ as follows: *y* = *X*_*c*_*β*_*cvc*_ + *X*_*i*_*β*_*ivc*_ + *u*_*vc*_ + *ϵ*_*vc*_, where *vc* indicates the variance components. To estimate the variance components, we must again assume that $Var\left({u}_{vc}\right)={\sigma}_g^2K$ and $Var\left({\epsilon}_{vc}\right)={\sigma}_e^2I$, such that $Var(y)={\sigma}_g^2K+{\sigma}_e^2I$. The EMMA technique can then be used to estimate the variance components ${\sigma}_g^2$ and ${\sigma}_e^2$ as well as a matrix *B* (and its inverse) such that $B{B}^{\prime }=H=\frac{Var(y)}{\sigma_g^2}=K+\frac{\sigma_e^2}{\sigma_g^2}I$. Thereafter, for every marker (*k*) we can compute (as an EMMAX-type approximation) the full and reduced models as: *B*^−1^*y* = *B*^−1^*X*_*c*_*β*_*kc*_ + *B*^−1^*X*_*i*_*β*_*ki*_ + *B*^−1^*X*_*k*_*β*_*kp*_ + *B*^−1^*X*_*ip*_*β*_*ip*_ + *B*^−1^(*u*_*full*_ + *ϵ*_*full*_) for the full model, where *B*^−1^(*u*_*full*_ + *ϵ*_*full*_) is assumed to be an error term proportional to the identity matrix, and as *B*^−1^*X*_*c*_*β*_*krc*_ + *B*^−1^*X*_*i*_*β*_*kri*_ + *B*^−1^*X*_*k*_*β*_*rkp*_ + *B*^−1^(*u*_*reduced*_ + *ϵ*_*reduced*_) for the reduced model, where *B*^−1^(*u*_*reduced*_ + *ϵ*_*reduced*_) is assumed to be an error term proportional to the identity matrix. To estimate the significance of the full versus reduced model, an *F*-test was performed; with all analyses utilizing the EMMAX method [28, 29] (i.e., GWAAs, GxE GWAAs) produced and further evaluated by constructing Manhattan plots within SVS v8.8.2 (Golden Helix, Bozeman, MT). Moreover, while SVS explicitly computes the full model mentioned above and outputs all of its *β* values, it only performs an optimization of the reduced model computation, which is sufficient to determine the SSE of the reduced-model equation, and thereafter, estimate the full versus reduced model *P*-value via *F*-test. This optimization is used to solve: *MB*^−1^*y* = *MB*^−1^*X*_*k*_*β*_*rkp*_ + *ϵ*_*MB*_, where *M* = (*I* − *QQ*′), and *Q* is derived from performing the QR algorithm, as *QR* = *B*^−1^ [*X*_*c*_ ∣ *X*_*i*_]. All Gelbvieh QTL were defined by ≥ 2 SNP loci with MAF ≥ 0.01 (i.e., a lead SNP plus at least one additional supporting SNP within 1 Mb) which also met a nominal significance threshold (*P* ≤ 1e-05) [30, 31], and all EMMAX marker-based pseudo-heritability estimates were produced as previously described [28–30, 99, 100].

Genomic inflation factors (λ) for all analyses (GEMMA; EMMAX) were estimated from the observed and expected *P*-values using genABEL [103], and the relationships between the observed *P*-values were estimated (GEMMA versus EMMAX) via correlation coefficients (i.e., Pearson, Spearman) in R v3.3.3 [89].

## Supplementary information

**Additional file 1: Figure S1.** EMMAX birth weight (BW) analysis. **Figure S2.** EMMAX weaning weight (WW) analysis**. Figure S3.** EMMAX yearling weight (YW) analysis. **Figure S4.** EMMAX birth weight (BW) genotype-by-environment (GxE) analysis**. Figure S5.** EMMAX weaning weight (WW) genotype-by-environment (GxE). **Figure S6.** EMMAX yearling weight (YW) genotype-by-environment (GxE) analysis. **Table S1.** Summary of QTL detected by EMMAX for BW in U.S. Gelbvieh cattle. **Table S2.** Genomic inflation factors (λ) calculated using observed *P*-values and expected *P*-values. **Table S3.** Correlation coefficients for GEMMA versus EMMAX *P*-values. **Table S4.** Summary of QTL detected by EMMAX for WW in U.S. Gelbvieh cattle. **Table S5.** Summary of QTL detected by EMMAX for YW in U.S. Gelbvieh cattle. **Table S6.** Summary of GxE QTL detected by EMMAX for BW in U.S. Gelbvieh cattle.**Table S7.** Summary of GxE QTL detected by EMMAX for YW in U.S. Gelbvieh cattle.

**Additional file 2.** Summary of lead and supporting SNPs from analyses for BW, WW, YW, BW GxE, WW GxE, and YW GxE using 778K imputed genotypes, including QTL chromosome, base pair, and rounded Mb.

**Additional file 3.** Summary of SNP panels used in analyses, including number of SNPs and individuals available before and after filtering.

## Data Availability

Data are available for non-commercial use via data use agreement (DUA) with the American Gelbvieh Association.

## Abbreviations

BW: Birth Weight
GWAA: Genome-wide association analysis
GxE: Genotype-by-environment interaction
QTL: Quantitative Trait Locus
WW: Weaning Weight
YW: Yearling Weight
