# Supplementary material for: Genome-wide association and genotype by environment interactions for growth traits in U.S. Gelbvieh cattle
Source: BMC Genomics. 2019 Dec 4;20:926. doi: 10.1186/s12864-019-6231-y (PMC6892214; doi:10.1186/s12864-019-6231-y)
Supplement: Supplementary file 1 — Additional file 1: Figure S1. EMMAX birth weight (BW) analysis. Figure S2. EMMAX weaning weight (WW) analysis. Figure S3. EMMAX yearling weight (YW) analysis. Figure S4. EMMAX birth weight (BW) genotype-by-environment (GxE) analysis. Figure S5. EMMAX weaning weight (WW) genotype-by-environment (GxE). Figure S6. EMMAX yearling weight (YW) genotype-by-environment (GxE) analysis. Table S1. Summary of QTL detected by EMMAX for BW in U.S. Gelbvieh cattle. Table S2. Genomic inflation factors (λ) calculated using observed P-values and expected P-values. Table S3. Correlation coefficients for GEMMA versus EMMAX P-values. Table S4. Summary of QTL detected by EMMAX for WW in U.S. Gelbvieh cattle. Table S5. Summary of QTL detected by EMMAX for YW in U.S. Gelbvieh cattle. Table S6. Summary of GxE QTL detected by EMMAX for BW in U.S. Gelbvieh cattle.Table S7. Summary of GxE QTL detected by EMMAX for YW in U.S. Gelbvieh cattle. [file 12864_2019_6231_MOESM1_ESM.docx]

**Supplemental Information**

**Figure S1. EMMAX birth weight (BW) analysis.**  The top pane reveals a Manhattan plot of –log_10_ P-values from EMMAX analysis, whereas the bottom pane reflects the estimated proportion of variance explained (PVE) by marker effects for n = 10,837 Gelbvieh cattle. A summary of all markers passing the nominal significance threshold [31] are presented in Table S1.

**Figure S2. EMMAX weaning weight (WW) analysis.** The top pane reveals a Manhattan plot of –log_10_ P-values from EMMAX analysis, whereas the bottom pane reflects the estimated proportion of variance explained (PVE) by marker effects for n = 10,837 Gelbvieh cattle. A summary of all markers passing the nominal significance threshold [31] are presented in Table S4.

**Figure S3. EMMAX yearling weight (YW) analysis.**  The top pane reveals a Manhattan plot of –log_10_ P-values from EMMAX analysis, whereas the bottom pane reflects the estimated proportion of variance explained (PVE) by marker effects for n = 10,837 Gelbvieh cattle. A summary of all markers passing the nominal significance threshold [31] are presented in Table S5.

**Figure S4. EMMAX birth weight (BW) genotype-by-environment (GxE) analysis.**  The top pane reveals a Manhattan plot of –log_10_ P-values from EMMAX analysis, whereas the bottom pane reflects the estimated proportion of variance explained (PVE) by marker effects for n = 10,837 Gelbvieh cattle. A summary of all markers passing the nominal significance threshold [31] are presented in Table S6.

**Figure S5. EMMAX weaning weight (WW) genotype-by-environment (GxE).** The top pane reveals a Manhattan plot of –log_10_ P-values from EMMAX analysis, whereas the bottom pane reflects the estimated proportion of variance explained (PVE) by marker effects for n = 10,837 Gelbvieh cattle. No QTL signals met our criteria for reporting (see Methods).

**Figure S6. EMMAX yearling weight (YW) genotype-by-environment (GxE) analysis.** The top pane reveals a Manhattan plot of –log_10_ P-values from EMMAX analysis, whereas the bottom pane reflects the estimated proportion of variance explained (PVE) by marker effects for n = 10,837 Gelbvieh cattle. A summary of all markers passing the nominal significance threshold [31] are presented in Table S7.

**Table S1.** Summary of QTL detected by EMMAX for BW in U.S. Gelbvieh cattle.

| ***Chr_Mb*** | ***EMMAX***  ***-log_10_***  ***P-value*** | ***Regression***  ***Beta*** | ***PVE*** | ***Supporting***  ***SNPs*** | ***Positional***  ***Candidate***  ***Genes*** | ***Lead SNP***  ***Location*** | ***Scientific Precedence [reference]; organism; trait*** |
| --- | --- | --- | --- | --- | --- | --- | --- |
| ***6_39*** | 29.66 | -1.795 | 0.013 | 152 | *NCAPG* | Exon^a^ | [18, 21, 30, 35–39]; Cattle, chicken; stature, calving ease and growth traits association, SimAngus mid-test metabolic weight association, fetal growth, carcass trait association, average daily gain and daily feed intake, muscle mass |
| ***14_25*** | 28.93 | -1.623 | 0.012 | 67 | *PLAG1* | 3’UTR | [2, 14, 18, 21, 30, 32–34]; Cattle; SimAngus mid-test metabolic weight association, carcass weight, stature, body weight and milk |
| ***14_26*** | 14.32 | -1.037 | 0.006 | 52 | *IMPAD1,*  *FAM110B* | Intergenic | [30, 32, 34, 40]; Cattle; SimAngus mid-test metabolic weight association, carcass weight association, stature and body weight association, bone and cartilage system |
| ***14_24*** | 12.83 | -0.947 | 0.005 | 49 | *RP1, XKR4* | Intergenic | [2, 30, 43, 44, 57]; Cattle; birth weight association, SimAngus mid-test metabolic weight association, growth trait association, feed intake and growth traits, retina development |
| ***6_42*** | 12.65 | -1.266 | 0.005 | 27 | *KCNIP4* | Intron | [39, 41, 42]; Chicken, cattle, human; growth and muscle mass trait association, potassium channel activity |
| ***6_38*** | 10.34 | -1.096 | 0.004 | 49 | *HERC6,*  *PPM1K* | Intergenic | [49, 58, 59]; Cattle; milk, fat, and protein yield, metabolic processes, feed efficiency association |
| ***6_41*** | 8.97 | 0.944 | 0.004 | 23 | *LOC782905,*  *SLIT2* | Intergenic | [39, 49–53]; Cattle, chicken, human; milk fat and protein association, organ and muscle weight, development of central nervous system, tumor suppressor activity |
| ***7_93*** | 8.65 | 0.952 | 0.003 | 31 | *LOC101905238, ARRDC3* | Intergenic | [14, 22, 30, 46]; Cattle; body and carcass weight association, calving ease, average daily gain in Hereford, growth and muscularity, birth weight, weaning weight, yearling weight, and ribeye area in Angus |
| ***20_05*** | 8.13 | 0.925 | 0.003 | 17 | *LOC104975192,*  *STC2* | Intergenic | [30, 45]; Cattle, mouse; mid-test metabolic weight in Hereford and SimAngus, developing and adult tissue maintenance, body size, related to post-natal growth |
| ***6_40*** | 7.49 | -0.852 | 0.003 | 7 | *LCORL,*  *LOC782905* | Intergenic | [18, 21, 37–39, 50, 55, 56]; Cattle, sheep; stature, muscle and organ growth, feed intake and gain association, growth and carcass traits, skeletal growth and muscle mass |
| ***6_34*** | 6.63 | -1.591 | 0.003 | 8 | *LOC104972717, LOC526089* | Intergenic | NA |
| ***6_36*** | 6.44 | -0.782 | 0.003 | 15 | *CCSER1* | Intron | [14, 60]; Cattle, human; body and carcass weight association, regulator of mitosis |
| ***14_23*** | 5.89 | 0.584 | 0.002 | 3 | *ST18* | Intron | [54]; Human; regulation of apoptosis and inflammatory response |
| ***6_29*** | 5.09 | -0.602 | 0.002 | 2 | *LOC530285, LOC100296313* | Intergenic | NA |

^a^ Indicates a predicted nonsynonymous mutation Ile🡪Met, exon 9

**Table S2.** Genomic inflation factors (λ) calculated using observed *P*-values and expected *P*-values.

|  | **GEMMA λ** | **GEMMA SE of λ** | **EMMAX λ** | **EMMAX SE of λ** |
| --- | --- | --- | --- | --- |
| **BW** | 1.16 | 1.06E-03 | 1.14 | 7.10E-04 |
| **WW** | 1.09 | 6.38E-04 | 1.08 | 3.87E-04 |
| **YW** | 1.11 | 7.35E-04 | 1.12 | 4.92E-04 |
| **BW GxE** | 1.07 | 1.72E-05 | 0.78 | 1.12E-04 |
| **WW GxE** | 1.00 | 1.60E-05 | 0.77 | 1.06E-04 |
| **YW GxE** | 1.06 | 1.81E-05 | 0.62 | 1.26E-04 |

**Table S3.** Correlation coefficients for GEMMA versus EMMAX *P*-values.

|  | **Pearson Correlation** | **Spearman Correlation** |
| --- | --- | --- |
| **BW** | 0.95 | 0.95 |
| **WW** | 0.80 | 0.80 |
| **YW** | 0.95 | 0.95 |
| **BW GxE** | 0.34 | 0.34 |
| **WW GxE** | 0.32 | 0.31 |
| **YW GxE** | 0.37 | 0.37 |

**Table S4.** Summary of QTL detected by EMMAX for WW in U.S. Gelbvieh cattle.

| ***Chr_Mb*** | ***EMMAX***  ***-log_10_***  ***P-value*** | ***Regression Beta*** | ***PVE*** | ***Supporting SNPs*** | ***Positional Candidate Genes*** | ***Lead SNP Location*** | ***Scientific Precedence [reference]; organism; trait*** |
| --- | --- | --- | --- | --- | --- | --- | --- |
| ***6_39*** | 20.43 | -11.205 | 0.008 | 112 | *NCAPG* | Exon^a^ | [18, 21, 30, 35–39]; Cattle, chicken; stature, calving ease and growth traits association, SimAngus mid-test metabolic weight association, fetal growth, carcass trait association, average daily gain and daily feed intake, muscle mass |
| ***14_25*** | 10.77 | -7.299 | 0.004 | 8 | *PLAG1* | 3’UTR | [2, 14, 18, 21, 30, 32–34]; Cattle; SimAngus mid-test metabolic weight association, carcass weight, stature, body weight and milk |
| ***6_36*** | 9.32 | -7.686 | 0.004 | 32 | *CCSER1* | Intron | [14, 60]; Cattle, human; body and carcass weight association, regulator of mitosis |
| ***6_42*** | 9.31 | -7.191 | 0.004 | 5 | *KCNIP4* | Intron | [39, 41, 42]; Chicken, cattle, human; growth and muscle mass trait association, potassium channel activity |
| ***6_38*** | 8.66 | -7.574 | 0.003 | 14 | *HERC6, PPM1K* | Intergenic | [49, 58, 59]; Cattle; milk, fat, and protein yield, metabolic processes, feed efficiency association |
| ***14_26*** | 8.37 | -5.655 | 0.003 | 16 | *IMPAD1, FAM110B* | Intergenic | [30, 32, 34, 40]; Cattle; SimAngus mid-test metabolic weight association, carcass weight association, stature and body weight association, bone and cartilage system |
| ***6_37*** | 7.74 | -6.556 | 0.003 | 6 | *SNCA, GPRIN3* | Intergenic | [61–64]; Human, goat, equine; neurological regulation, milk and meat associations, tendon tissue association |
| ***5_60*** | 7.27 | 9.486 | 0.003 | 5 | *LOC511753, LOC527216* | Intergenic | NA |
| ***6_41*** | 7.23 | -6.294 | 0.003 | 6 | *LOC782905, SLIT2* | Intergenic | [39, 49–53]; Cattle, chicken, human; milk fat and protein association, organ and muscle weight, development of central nervous system, tumor suppressor activity |
| ***6_31*** | 7.08 | -6.583 | 0.003 | 7 | *UNC5C* | 3’UTR | [65, 66]; Cattle; axon migration and embryonic development |
| ***20_05*** | 6.98 | 5.211 | 0.003 | 3 | *ERGIC1* | Intron | [14, 30]; Cattle; transport between endoplasmic reticulum and golgi, SimAngus and Hereford mid-test metabolic weight test association |
| ***6_34*** | 6.53 | 5.690 | 0.002 | 6 | *LOC100336621, LOC104972717* | Intergenic | NA |
| ***14_24*** | 5.52 | -5.237 | 0.002 | 4 | *XKR4* | Intron | [2, 30, 43, 44]; Cattle; birth weight association, SimAngus mid-test metabolic weight association, growth trait association, feed intake and growth traits |
| ***28_37*** | 5.16 | 4.348 | 0.002 | 2 | *SH2D4B, LOC787215* | Intergenic | [67]; Mouse; immunological response |

^a^ Indicates a predicted nonsynonymous mutation Ile🡪Met, exon 9

**Table S5.** Summary of QTL detected by EMMAX for YW in U.S. Gelbvieh cattle.

| **Chr_Mb** | ***EMMAX***  ***-log_10_***  ***P-value*** | ***Regression Beta*** | ***PVE*** | ***Supporting SNPs*** | ***Positional Candidate Genes*** | ***Lead SNP Location*** | ***Scientific Precedence [reference]; organism; trait*** |
| --- | --- | --- | --- | --- | --- | --- | --- |
| **6_39** | 25.13 | -24.287 | 0.013 | 108 | *LCORL* | Intron | [18, 21, 30, 37–39, 55, 56]; Cattle, sheep; stature, SimAngus mid-test metabolic weight association, muscle and organ growth, feed intake and gain association, growth and carcass traits, skeletal growth and muscle mass |
| **14_25** | 13.91 | -15.418 | 0.007 | 3 | *PLAG1* | 3’UTR | [2, 14, 18, 21, 30, 32–34]; Cattle; SimAngus mid-test metabolic weight association, carcass weight, stature, body weight and milk |
| **6_38** | 13.43 | -17.747 | 0.007 | 53 | *HERC6, PPM1K* | Intergenic | [49, 58, 59]; Cattle; milk, fat, and protein yield, metabolic processes, feed efficiency association |
| **6_42** | 13.38 | -16.123 | 0.007 | 26 | *KCNIP4* | Intron | [39, 41, 42]; Chicken, cattle, human; growth and muscle mass trait association, potassium channel activity |
| **6_37** | 12.66 | -15.728 | 0.007 | 34 | *SNCA, GPRIN3* | Intergenic | [61–64]; Human, goat, equine; neurological regulation, milk and meat associations, tendon tissue association |
| **6_36** | 10.10 | -14.441 | 0.005 | 32 | *CCSER1* | Intron | [14, 60]; Cattle, human; body and carcass weight association, regulator of mitosis |
| **6_41** | 9.86 | -13.955 | 0.005 | 26 | *SLIT2* | Intron | [39, 49–53]; Cattle, chicken, human; milk fat and protein association, organ and muscle weight, development of central nervous system, tumor suppressor activity |
| **5_60** | 8.57 | 19.910 | 0.004 | 8 | *LOC511753, LOC527216* | Intergenic | NA |
| **14_26** | 8.45 | -11.412 | 0.004 | 20 | *IMPAD1, FAM110B* | Intergenic | [30, 32, 34, 40]; Cattle; SimAngus mid-test metabolic weight association, carcass weight association, stature and body weight association, bone and cartilage system |
| **6_40** | 7.12 | -15.870 | 0.004 | 18 | *LOC782905, SLIT2* | Intergenic | [39, 49–53]; Cattle, chicken, human; milk fat and protein association, organ and muscle weight, development of central nervous system, tumor suppressor activity |
| **14_24** | 7.12 | 9.920 | 0.003 | 2 | *RP1, XKR4* | Intergenic | [2, 30, 43, 44, 57]; Cattle; birth weight association, SimAngus mid-test metabolic weight association, growth trait association, feed intake and growth traits, retina development |
| **14_27** | 6.90 | 10.122 | 0.003 | 7 | *NSMAF* | Intron | [30, 68]; Cattle, human; Angus residual feed intake, immune system response |
| **7_93** | 6.88 | 11.644 | 0.003 | 15 | *LOC101905238, ARRDC3* | Intergenic | [14, 22, 30, 46]; Cattle; body and carcass weight association, calving ease, average daily gain in Hereford, growth and muscularity, birth weight, weaning weight, yearling weight, and ribeye area in Angus |
| **6_45** | 6.26 | -9.356 | 0.003 | 4 | *PPARGC1A* | Intron | [70, 71]; Cattle; milk composition, milk fat synthesis, and yield |
| **6_29** | 5.60 | -10.847 | 0.003 | 5 | *LOC782977, LOC530285* | Intergenic | NA |

**Table S6.** Summary of GxE QTL detected by EMMAX for BW in U.S. Gelbvieh cattle.

| ***Chr_Mb*** | ***EMMAX***  ***-log_10_***  ***P-value*** | ***Regression Beta*** | ***PVE*** | ***Supporting SNPs*** | ***Positional Candidate Genes*** | ***Lead SNP Location*** | ***Scientific Precedence [reference]; organism; trait*** |
| --- | --- | --- | --- | --- | --- | --- | --- |
| ***13_67*** | 5.17 | 0.641 | 0.003 | 2 | *CTNNBL1* | 5’UTR | [76]; Human; obesity association |

**Table S7.** Summary of GxE QTL detected by EMMAX for YW in U.S. Gelbvieh cattle.

| ***Chr_Mb*** | ***EMMAX***  ***-log_10_***  ***P-value*** | ***Regression Beta*** | ***PVE*** | ***Supporting SNPs*** | ***Positional Candidate Genes*** | ***Lead SNP Location*** | ***Scientific Precedence [reference]; organism; trait*** |
| --- | --- | --- | --- | --- | --- | --- | --- |
| ***5_116*** | 6.71 | 3.635 | 0.005 | 4 | *PHF21B* | Intron | [86, 87]; Human, rat; modulates stress response, cell division regulation |
